# Determinants of Phagosomal pH During Host-Pathogen Interactions

**DOI:** 10.3389/fcell.2020.624958

**Published:** 2021-01-11

**Authors:** Johannes Westman, Sergio Grinstein

**Affiliations:** ^1^Program in Cell Biology, The Hospital for Sick Children, Toronto, ON, Canada; ^2^Department of Biochemistry, University of Toronto, Toronto, ON, Canada

**Keywords:** pH, phagocytosis, macrophage, pathogen, V-ATPase, proton, phagosome acidification, ion transport

## Abstract

The ability of phagosomes to halt microbial growth is intimately linked to their ability to acidify their luminal pH. Establishment and maintenance of an acidic lumen requires precise co-ordination of H^+^ pumping and counter-ion permeation to offset the countervailing H^+^ leakage. Despite the best efforts of professional phagocytes, however, a number of specialized pathogens survive and even replicate inside phagosomes. In such instances, pathogens target the pH-regulatory machinery of the host cell in an effort to survive inside or escape from phagosomes. This review aims to describe how phagosomal pH is regulated during phagocytosis, why it varies in different types of professional phagocytes and the strategies developed by prototypical intracellular pathogens to manipulate phagosomal pH to survive, replicate, and eventually escape from the phagocyte.

## Introduction to Phagocytosis

Innate immune cells such as macrophages, neutrophils and dendritic cells (DC) carry out an astounding variety of functions, ranging from killing invading pathogens to cytokine production, antigen presentation, and tissue homeostasis. Phagocytosis, an essential response of these cells –collectively termed professional phagocytes– is an elegant and complex process involving the recognition, engulfment, and degradation of foreign or dead material, which has unquestionable importance in innate immunity. Phagocytes are endowed with an assortment of receptors that initiate the internalization of diverse target particles. Some phagocytic receptors recognize endogenous patterns on the surface of the foreign target, while others identify their prey indirectly by interacting with opsonins (serum components) bound to the target’s surface (Flannagan et al., 2012). Independently of the receptors and ligands involved, their interaction spawns an intricate intracellular signaling system culminating in the protrusion of the plasma membrane around the target, leading to its complete envelopment and internalization (Niedergang and Grinstein, 2018). After internalization, phagosomes acquire microbicidal and degradative capacity by a graded process called phagosome maturation. The nascent phagosome sequentially fuses with early endosomes, late endosomes, and ultimately, lysosomes, leading to the accrual of vacuolar proton (H^+^)-pumping ATPases (V-ATPases), NADPH oxidase, and a variety of microbicidal peptides and degradative enzymes (Flannagan et al., 2012). Regulation of the phagosomal pH is of importance for most phagosomal functions. V-ATPases –that are normally enriched in late endosomes and lysosomes– are gradually acquired by phagosomes during the course of their maturation and are the source of their luminal acidification; they utilize energy derived from ATP hydrolysis to pump cytosolic H^+^ into the phagosome lumen. Regulation of the phagosomal pH is, however, an intricate process, also depending on the permeability to counter-ions and the “leak” of H^+^ equivalents. Why these events are of high importance, how they are strictly regulated, and how pathogens share strategies to subvert phagosomal pH are further discussed in this review.

## Why Is There a Need for Phagosomal PH Regulation?

Phagosomal acidification is a hallmark of phagosome maturation, and the progressive luminal acidification results from the gradual increase of active V-ATPases. Various key functions of professional phagocytes require phagosomal acidification. For example, phagocytic receptors, including Fcγ receptors, integrins, and C-type lectin receptors such as dectin-1, are initially internalized along with the prey but need to be recycled to the cell surface. pH alters the affinity of the interaction between the phagocytic receptors and their ligands. The intraphagosomal acidification enables the dissociation and recycling of phagocytic receptors back to the plasmalemma (Buckley et al., 2016).

**FIGURE 1:**
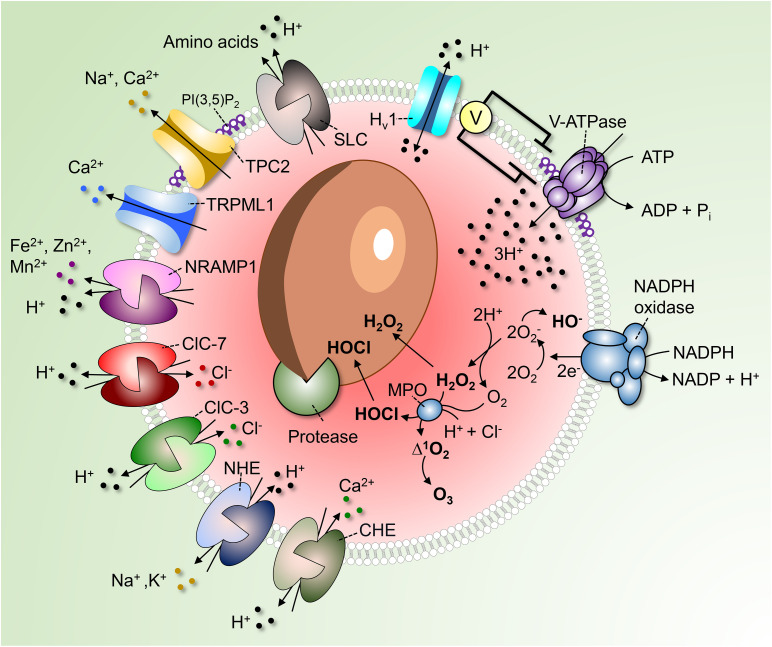
Regulation of phagosomal pH in phagocytes. V-ATPases hydrolyze ATP to pump 3H^+^ into the phagosome. As the V-ATPase is electrogenic, its continued operation is dependent on parallel counter-ion influxes. These can be provided by rheogenic anion antiporters like ClC-7 and ClC-3 and/or through cation efflux via channels such as TPC2 and TRPML1. Conversion of PI(3)P into PI(3,5)P_2_ by PIKfyve regulates TRPML1, TCP-2 and possibly also the V-ATPase itself. A number of pathways promote the “leakage” of H^+^ out of phagosomes. These include monovalent and divalent cation/H^+^ antiporters (NHE and CHE) or symporters (NRAMP1), Hv1 H^+^-selective channels and amino acid-H^+^ cotransporters of the SLC family. In addition, H^+^ is consumed during antimicrobial activities by products of the NADPH oxidase to produce hydroxyl radicals, HOCl, and H_2_O_2_. Low luminal pH is required for the autoactivation of various phagosomal proteases and for protonation of microbicidal effectors.

Perhaps more apparent is the role of phagosomal acidification in facilitating the microbicidal response. Low pH is critical for the converting of zymogens, such as nucleases, lipases, and proteases, to their active, degradative form. For example, cathepsin B is delivered to immature phagosomes as pro-cathepsin B. Progression to the more acidic mature phagosome (or phagolysosome) is accompanied by a conformational change of the pro-cathepsin B followed by its autoactivation and cleavage, yielding the mature enzyme (Pungercar et al., 2009). Additionally, the NADPH oxidase requires H^+^ to generate hydrogen peroxide (H_2_O_2_), an important microbicidal reactive oxygen species (ROS), inside the phagosome (Figure 1). Superoxide anions (O_2_^–^), the primary product of the NADPH oxidase, are spontaneously dismutated into H_2_O_2_ in an acidic environment, consuming luminal H^+^ (Nauseef, 2019; Figure 1). Along with chloride (Cl^–^), H^+^ are also required for the generation of hypochlorous acid (HOCl) from H_2_O_2_ by phagosomal myeloperoxidase (MPO) (Figure 1). HOCl is yet another powerful antimicrobial agent, and similar to H^+^, a deficient supply of this anion in phagosomes is linked to defects in innate immunity (Wang, 2016).

Phagocytes can also exert microbiostatic or microbicidal effects by limiting the macro- and micro-nutrients available to the ingested pathogen while inside the phagosome. To limit intraphagosomal microbial replication, the membrane-associated transporter of divalent metal cations natural resistance-associated macrophage protein 1 (NRAMP1, also known as SLC11A1) depletes the phagosome of Fe^2+^, Mn^2+^, and Mg^2+^ (Figure 1). The force driving the efflux of these essential metal ions is the transmembrane H^+^ gradient generated by the V-ATPase. Attesting to its importance, mutations in NRAMP1 impair the resistance of mice to infections with intracellular pathogens such as *Salmonella, Leishmania*, and *Mycobacterium* (Govoni and Gros, 1998), and susceptibility to leprosy is linked to the human NRAMP1 gene (Abel et al., 1998). Luminal H^+^ are likewise crucial for nutrient absorption after microbial killing. For example, amino acids derived from the degradation of dead microbial or apoptotic cells are transported out of the phagolysosome into the cytosol via H^+^-coupled solute carrier (SLC) transporters (Figure 1).

Protonation of microbial components in the acidic phagosomal lumen will, in some cases, stimulate acid stress responses of the microorganism, which can either facilitate or impair their ability to survive intracellularly (Guan and Liu, 2020).

Lastly, precise regulation of the phagosomal pH is also required for optimal antigen presentation, an essential reaction linking the innate and acquired immune systems. DCs, the quintessential antigen-presenting cells, must degrade incoming proteins to generate peptides suitable for presentation to lymphoid cells. Unrestrained degradation, however, will yield inappropriately small peptides; thus, an intermediate phagosomal pH is required to prevent excessive proteolysis of antigens prior to loading onto MHC class II glycoproteins.

## How Is Phagosomal pH Regulated?

Phagocytosis can be conceptually divided into two events: phagosome formation and phagosome maturation. Phagosome formation refers to the binding of the target particle followed by the actin-dependent extension of pseudopodia and lamellipodia that are often circular that encircle and ultimately trap the prey in a sealed vacuole, the nascent phagosome (see review (Niedergang and Grinstein, 2018) for how to build a phagosome). Immediately after closure and scission, the nascent phagosome’s luminal pH reflects the extracellular pH, as the bulk fluid inside the lumen originates from the surrounding milieu. As stated earlier, a gradual maturation process ensues, whereby early endosomes, late endosomes, and lysosomes fuse with phagosomes. Phosphoinositides, Rab GTPases, soluble N-ethylmaleimide-sensitive-factor attachment protein receptors (SNAREs) and coat/tubulating proteins dictate maturation by signaling and mediating fusion and fission events at the phagosomal membrane (Fairn and Grinstein, 2012). These events ensure that the phagosomal membrane and luminal contents transition into a degradative and microbicidal structure, while maintaining its size approximately constant. Fusion of the late phagosome with lysosomes is a fundamental event. This is the stage where most of the V-ATPases are acquired, an event accompanied by the profound luminal acidification that reaches pH ≤ 5 (Levin et al., 2016). V-ATPases, are large, multi-subunit complexes that convert chemical energy stored in ATP into mechanically driven H^+^ translocation (Forgac, 2007). Clearly, the number of V-ATPases inserted into the phagolysosomal membrane will be an important determinant of the rate and extent luminal acidification. However, not all the V-ATPases will be equally active at all times and the factors controlling the rate of pumping need to be considered (Maxson and Grinstein, 2014). Remarkably, little is known about the regulation of mammalian V-ATPases. Their activity is affected by the lipid composition of the lysosomal membrane: sphingolipids are required for optimal ATP hydrolysis, and lack of these sphingolipids results in impaired acidification (Chung et al., 2003). Moreover, the phosphoinositide PtdIns(3,5)P_2_, which has gotten much attention lately as a regulator of phagosomal ion channels, is suggested to be required for acidification. At least in some systems, absence of PtdIns(3,5)P_2_ is associated with decreased V-ATPase activity and H^+^ pumping rate (Li et al., 2014).

Active V-ATPases transport 3H^+^ per ATP hydrolyzed; the V-ATPases deliver H^+^ across the membrane unaccompanied by other ions and, as such, are electrogenic. It follows that counter-ion fluxes must occur in parallel to enable measurable changes in pH; in their absence, an electrical potential would be generated that would oppose significant H^+^ flux. The source of counter-ion fluxes that neutralize the electrogenic H^+^ flux is uncertain; one promising candidate is the influx of Cl^–^ via channels or rheogenic antiporters (Kornak et al., 2001; Di et al., 2006; Graves et al., 2008). To our knowledge, counter-ion fluxes have not been directly analyzed in phagolysosomes. However, as the phagolysosomal membrane resembles that of lysosomes, it is reasonable to assume that H^+^ fluxes are similarly regulated. Therefore, extrapolation of the knowledge obtained from lysosomes seems warranted. Some Cl^–^ conductive pathways function as *bona fide* channels: these include Cl^–^ intracellular channels, cystic fibrosis transmembrane conductance regulator (CFTR), and the volume-regulated anion channel (VRAC or LRRC8) plasmalemmal regulated channels, the presence and operation of which in lysosomes is still debated. Others, originally thought to be conductive channels, were subsequently found to operate as antiporters: members of the Cl^–^ channel (ClC) family mediate the uptake of 2 Cl^–^ ions in exchange for a luminal H^+^. The resulting charge imbalance renders these antiporters rheogenic, effectuating the net uptake of one negative charge when operating in their “normal” forward direction. Some members of the ClC family, namely, ClC-4, ClC-5, and ClC-6 are found in early compartments of the endosomal pathway, whereas ClC-3 is found in early/late endosomes, and ClC-7 predominantly localizes to lysosomes (Kornak et al., 2001; Wang, 2016), and therefore most likely also to phagolysosomes. At first glance, operation of ClC-7 as a 2Cl^–^/H^+^ antiport would appear counterproductive, as it tends to decrease the accumulation of luminal H^+^ that is the objective of the V-ATPases. However, it should be borne in mind that exchange of 2 cytosolic Cl^–^ for one luminal H^+^ results in the net intraphagosomal gain of 3 negative charge equivalents, and that V-ATPases translocate 3H^+^ per functional cycle. Thus, sacrificing the loss of one H^+^ to enable the neutralization of the two net H^+^ gained appears justified.

The role of ClC-7 in establishing the acidification of lysosomes (and presumably, by extension, of phagolysosomes) can best be established by knocking out the antiporters. This seemingly definitive and straightforward approach, however, has yielded contradictory results, with some authors reporting impairment of lysosomal acidification (Graves et al., 2008), while others found no significant effect (Steinberg et al., 2010; Weinert et al., 2010). It is clear, nevertheless, that the antiporter is required for the function of lysosomes in at least some specialized settings since mice deficient in ClC-7 display severe osteopetrosis and retinal degeneration presumably associated with improper endolysosomal acidification (Kornak et al., 2001). It is nevertheless puzzling that ClC-7 knockout mice show neurodegeneration and severe lysosomal storage disease without elevated lysosomal pH (Kasper et al., 2005).

Redundancy with other anion transporters could explain the apparent discrepancies reported in ClC-7-deficient animals. ClC-3 also functions as a late endo(lyso)somal 2Cl^–^/H^+^ exchanger. However, due to the strong voltage-dependence of ClC-3, luminal positive voltage or acidic phagosomal pH could shift the exchange mode to a conductive Cl^–^ channel (Matsuda et al., 2010; Stauber et al., 2012). On the other hand, an alternative –but not mutually exclusive– mechanism could counteract the V-ATPase’s electrogenic nature. Specifically, it has been suggested that an efflux of luminal cations (as opposed to the influx of anions) may serve to neutralize the electrogenicity of H^+^ pumping via the V-ATPase. Steinberg et al. (2010) investigated the role of both anion influx and cation efflux from lysosomes, assessing their individual contribution to luminal acidification. In their study, replacement of cytosolic Cl^–^ with impermeant anions did not significantly alter the rate of V-ATPase pumping. These authors found that lysosomes acidified similarly in CFTR- and ClC-7-deficient cells. Instead, they demonstrated that lysosomes required Na^+^ and K^+^ efflux for proper luminal acidification (Steinberg et al., 2010). A similar efflux of Na^+^ and K^+^ could support (phago)lysosomal acidification.

Besides Na^+^ and K^+^, (phago)lysosomes also store Ca^2+^ (Westman et al., 2019). Lysosomes are thought to accumulate Ca^2+^, at least in part, via Ca^2+^/H^+^ exchange; accordingly, V-ATPase inhibition using well-established pharmacological agents impairs Ca^2+^ loading. The Ca^2+^ accumulated by this ostensibly electroneutral exchange (two H^+^ are thought to exchange for each Ca^2+^) could in principle be released via conductive pathways, serving as a counter-ion. Lysosomal Ca^2+^ is predominantly released via TRPML1 and, to some extent, via two-pore channels (TPC) (Jin et al., 2020). Though not widely acknowledged in the literature, TPCs are considerably more permeable to Na^+^ than Ca^2+^ (Morgan and Galione, 2014; Guo et al., 2017). As such, these channels could provide the route for the efflux of monovalent cationic counter-ions needed to neutralize the electrogenic H^+^ pumping. Consistent with this notion, studies of lysosomal pH in macrophages from mice lacking TPC-1 and TPC-2 show that lysosomes exhibited elevated lysosomal pH under starvation (Cang et al., 2013).

Other divalent cations, including Zn^2+^, Cu^2+^ and Fe^2+^ are also transported across endosomes and lysosomes by carriers coupled to the H^+^ gradient, including the above mentioned NRAMP1 and also NRAMP2. While there has been some discrepancy regarding the mode of NRAMP coupling (i.e., co- vs. counter-transport), it is nevertheless clear that these transporters are essential for heavy metal homeostasis and for proper host responsiveness to pathogens (Forbes and Gros, 2001). In contrast, it is clear that the endomembrane Zn^2+^ transporters (ZnT) of the SLC30 family function as H^+^ antiporters (Baltaci and Yuce, 2018).

The concomitant efflux (“leak”) of H^+^ from (phago)lysosomes is another important determinant of their steady state pH. Multiple pathways contribute to the leak and some are probably unsuspected at present. Known pathways include the ClC-7 (2Cl^–^/H^+^), ClC-3 (2Cl^–^/H^+^), and CHE (Ca^2+^/2H^+^) antiporters mentioned above, as well as monovalent cation NHE (Na^+^ and or K^+^/H^+^) antiporters, H^+^-conductive channels such as the voltage-gated Hv1 (El Chemaly et al., 2014), and H^+^-coupled amino acid symporters. How active each of these systems is and how much they contribute to the regulation of pH is not at all clear, although they are collectively active at steady state. This is readily demonstrated by the alkalinization initiated immediately after inhibition of the V-ATPases by specific blockers like concanamycin or bafilomycin.

The acidifying effects of the V-ATPase are offset by H^+^ leakage, but also by H^+^ (equivalent) consumption by metabolic reactions occurring in the organellar lumen. Especially in neutrophils, which produce large amounts of ROS, H^+^ are consumed inside phagosomes in the course of O_2_^–^ dismutation and during the generation of H_2_O_2_ and HOCl. Hydrolytic reactions involved in cargo degradation are similarly likely to involve H^+^ consumption (Figure 1).

Lastly, it is worth mentioning that the lysosomal pH will inevitably be affected by changes in the pH of the surrounding cytosol. In this regard, it was recently reported that phagosomal acidification is dependent on the activity of the plasma membrane bicarbonate transporter SLC4A7, which determines the cytosolic pH. Knockout of SLC4A7 leads to cytosolic acidification and an associated impairment in phagosomal maturation, v-ATPase function, and acquisition of the NADPH oxidase (Sedlyarov et al., 2018).

## How Do Professional Phagocytes and Their Phagosomes Differ in pH Regulation?

Macrophages, DCs and neutrophils all internalize pathogens, apoptotic and necrotic debris with varying efficiency and for different purposes (Flannagan et al., 2012; Westman et al., 2020a). Depending on their localization and on environmental stimuli, phagocytes respond by undergoing cell polarization into distinct functional phenotypes. For macrophages these phenotypes can be divided into classically activated (M1-like) macrophages and alternatively activated (M2-like) macrophages. Naturally, macrophage polarization affects the properties of their phagosomes. M1-like macrophages (classically activated by LPS + IFNγ) are associated with engulfment and killing of pathogens, while M2-like macrophages (alternatively activated by IL-4) prioritize efferocytosis. The buffering capacity and H^+^ leakage permeability remain relatively unaltered between the two subtypes, which, however, show drastic changes in V-ATPase-dependent H^+^ pumping (Canton et al., 2014). In M1-like macrophages, the elimination of pathogens via NADPH oxidase activity is given priority at the expense of delayed acidification. Accordingly, M1-like macrophages show alkaline oscillations caused by H^+^ consumption upon O_2_^–^ dismutation, and the ROS generated delay the acquisition of V-ATPases. In contrast, M2-like macrophages, have reduced NADPH oxidase activity, rapidly acidify to clear apoptotic and necrotic debris.

Phagosomes formed by neutrophils are less acidic than those of macrophages and DCs (Nordenfelt and Tapper, 2011). Similar to phagosomes of M1-like polarized macrophages, neutrophil phagosomes are more alkaline for various reasons, all related to phagosomal ROS generation. Firstly, ROS production increases the permeability of the phagosomes, leading to increased H^+^ leak. Secondly, as in M1-like macrophages, O_2_^–^ dismutation associated with the robust NADPH oxidase activity consumes the majority of phagosomal H^+^. As the lumen remains neutral or even slightly alkaline, H^+^ enter the phagosome via voltage-gated Hv1 channels to facilitate the continuous production of high amounts of ROS. Lastly, H_2_O_2_ has been shown to impair V-ATPase recruitment to the neutrophil phagosome, further excluding it from the phagosome, which consequently decreases H^+^ influx (Jankowski et al., 2002; El Chemaly et al., 2014).

Phagocytosis plays a significantly different role in DCs compared to macrophages and neutrophils. Their primary role as professional antigen-presenting cells is to alert the immune system of the ongoing infection, rather than clearing the invading microorganisms. How DCs process antigenic epitopes directly affects the efficiency of their presentation to T cells (Delamarre et al., 2005). DCs sample the extracellular environment, engulf protein- and lipid-containing material, and process and present antigens to lymphocytes, which is essential for their differentiation, clonal expansion, and antibody production. In comparison to macrophages, the phagosomes of DCs acidify to a lower extent. Accordingly, phagosomes of DCs acquire lower amounts of the V-ATPase, and luminal H^+^ are continuously consumed by products of the NADPH oxidase. Moreover, the oxidase tightly regulates the level of proteolysis in the phagosomes of DCs. The consequence of the intermediate phagosomal pH and decreased protease activity is a more moderate digestion of epitopes, required for processing and presentation of microbial antigens (Mantegazza et al., 2008).

Monocytes circulating in the bloodstream supply peripheral tissues with monocyte-derived macrophages and DCs. Besides participating in the clearance of circulating platelets, monocytes can also internalize pathogens, a phenomenon that might play a protective role during disseminated bacterial infection and sepsis. Accordingly, certain monocytic populations increase their phagocytic activity during early stages of sepsis (Döring et al., 2015). Yet, remarkably little is known about how monocytes establish and regulate their phagosomal pH (Döring et al., 2015).

Cells other than mammalian innate immune cells can also ingest foreign particles. A well-studied example is *Dictyostelium discoideum*, a soil-dwelling ameba which feeds on bacteria (Dunn et al., 2018). Indeed, several important findings of phagocyte behavior have been unveiled studying *D. discoideum*, which has been used as a model system because many of its genes are homologous to human genes. As in mammalian phagocytes, the *D. discoideum* phagosome creates an antimicrobial environment via V-ATPase-dependent acidification, delivery of hydrolytic enzymes, generation of ROS, and regulation of metal ions fluxes (Dunn et al., 2018). The *D. discoideum* phagosome acidifies within 10–30 min by fusing with endo-lysosomes, that deliver V-ATPases (Clarke et al., 2002). Lysosomes of *D. discoideum* have been reported to acidify to ≤pH 3.5 (Marchetti et al., 2009) and it has therefore been suggested that their phagosomes could reach a comparable level of acidity. However, others report that *D. discoideum* phagosomes reach pH ≈5.0 (Rupper et al., 2001a,b; Sattler et al., 2013), similar to that of mammalian phagosomes. Following processing of their contents, *D. discoideum* phagosomes give rise to a post-lysosomal structure containing non-digestible bacterial remnants. Of note, this post-lysosome has a neutral pH as the V-ATPases, together with lysosomal enzymes, are removed for reuse by a mechanism involving the WASH complex (Carnell et al., 2011). The luminal contents of such post-lysosomes are expelled from the cells by exocytosis.

## How Do Pathogens Hijack Phagosomal pH?

**FIGURE 2:**
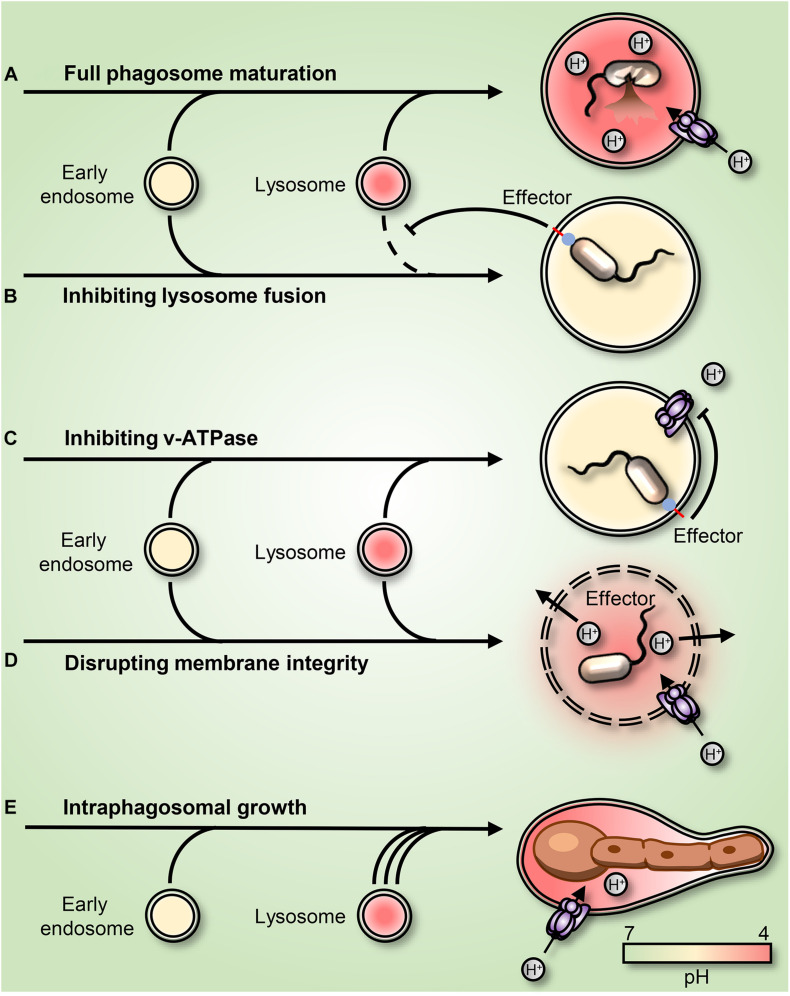
Strategies employed by pathogens to subvert or adapt to the acidic phagosomal pH. **(A)** Phagosome maturation leads to luminal acidification and subsequent killing of the internalized prey. **(B)** Mycobacteria and *Salmonella enterica* arrest phagosome maturation by impairing endo-lysosome insertion into the maturing phagosome. **(C)** *Legionella pneumophila* secretes into the host cytosol the effector sidK that binds and inhibits H^+^ pumping by the V-ATPase, leading to impaired acidification. **(D)** *Listeria monocytogenes* secretes the pore-forming toxin listeriolysin O and phospholipases, leading to a rupture of the phagosomal membrane. **(E)** *Candida albicans* survives and grows as filaments inside the acidic phagosome. The *C. albicans*-containing phagosome expands and remains acidic for hours before permanent rupture causes H^+^ leakage.

Even though phagocytes are efficient in killing most pathogens (Figure 2A), several species can survive and adapt after phagocytic uptake. Mechanisms for survival differ, but many unrelated pathogens focus their efforts on preventing phagosomal acidification. Some pathogens prevent the V-ATPase-dependent H^+^ accumulation by interfering with the endolysosomal fusion machinery (Figure 2B); others escape the phagosome. Inhibition of lysosomal fusion is a strategy shared by several distantly related bacteria including *Mycobacterium* spp. and *Salmonella* spp. (Buchmeier and Heffron, 1991; Russell, 2001). *M. tuberculosis* (Mtb) can arrest lysosomal insertion into phagosomes (at least partially) by producing the phosphatases SapM and MptpB. These target different phosphatidylinositol derivates that signal and direct phagosomal maturation (Walpole et al., 2018). Moreover, Mtb secretes the glycolipids phosphatidylinositol mannosides (PIM) and lipoarabinomannan (ManLAM), which redirect lipid-mediated trafficking by acting as decoys of mammalian lipids (Chua et al., 2004; Mishra et al., 2011). Mtb was also reported to regulate phagosomal acidification by interfering with the retention of the V-ATPases at the phagosomal surface (Wong et al., 2011). *S. enterica* employs a type III secretion system (TTSS) to inject bacterial effectors, including SopB, into the cytosol. Although the enzymatic activity of SopB is still under debate, it is clearly involved in the impairment of the maturation of the *Salmonell*a-containing phagosome (Norris et al., 1998; Hernandez et al., 2004; Mallo et al., 2008). *Shigella flexneri* similarly inserts effectors into its host cells using a TTSS. One of its main effectors, IpgD, is a homolog of *S. enterica* SopB and similarly dephosphorylates plasmalemmal and phagosomal phosphoinositides (Niebuhr et al., 2002).

Some pathogens survive in phagosomes by directly targeting the V-ATPase. *Legionella pneumophilia*, the causative agent of Legionnaires’ Disease, allows phagosomes to mature but not acidify. The bacterium produces several effectors to create a niche supportive of its replication. Amongst these is sidK, which *Legionella* secretes into the cytosol where it directly binds to the V-ATPase, inhibiting its function (Zhao et al., 2017; Figure 2C). *Listeria monocytogenes* escape phagosomes by secreting listeriolysin O and phospholipases (Smith et al., 1995; Nguyen et al., 2019; Figure 2D). Other intraphagosomal pathogens, such as *H. pylori*, Mtb, and *Candida albicans* have been reported to produce and secrete NH_3_, which in principle can bind to and buffer phagolysosomal H^+^, leading to phagosomal alkalinization (Schwartz and Allen, 2006; Song et al., 2011; Vylkova and Lorenz, 2014). Similarly, Mtb was proposed to release an antacid compound in an attempt to neutralize acidic phagosomes (Buter et al., 2019). However, the *C. albicans*-containing phagosome was recently shown to be permeable to NH_3_. Instead, it was demonstrated that *C. albicans* yeast convert into filaments that grow inside acidic phagosomes and that phagosomal alkalinization resulted from phagosomal H^+^ leakage caused by the associated mechanical strain (Westman et al., 2018). Whether the NH_3_-mediated buffering hypothesis applies in the cases of *H. pylori* and Mtb remains to be confirmed.

Other pathogens transcriptionally adapt to survive and grow within the microbicidal and nutrient-deprived environment of the phagosome. *C. albicans*, *Staphylococcus aureus*, and *Coxiella burnetti* all adapt to the microbicidal phagosome. Instead of interfering with phagosomal pH, these unrelated microorganisms use a convergent strategy, adapting metabolically to the nutrient-deprived environment inside acidic phagolysosomes (Lorenz et al., 2004; Voth and Heinzen, 2007; Flannagan et al., 2016). Like innate immune cells, engulfed microbes depend on amino acid/H^+^ symporters for nutrient acquisition and therefore require an inward H^+^ gradient. Most bacteria can pump excess H^+^ out of their cytoplasm to maintain pH homeostasis (Guan and Liu, 2020), and many manage to grow and even replicate within phagosomes. However, their intraphagosomal growth does not necessarily lead to phagosomal escape. In this regard, it was recently demonstrated that when subjected to the mechanical stress imposed by growing microorganisms, phagosomes have means to expand their surface area. This remarkable response is mediated by a secondary wave of lysosome insertion that maintains phagosome integrity and preserves the microbiostatic environment (Westman et al., 2020b; Figure 2E).

## Concluding Remarks and Future Directions

While the ability of phagosomes to acidify has been appreciated for more than a century, since Metchnikoff made his pioneering observations, the underlying determinants and its biological significance remain incompletely understood. The importance of the luminal acidification is highlighted by the convergent strategies developed by diverse pathogens to neutralize or bypass it. They appreciated these subtleties long before researchers did and developed means to manipulate the luminal pH to secure their survival and proliferation.

As should be apparent from this review, our understanding of phagosomal pH regulation and its role in immune function are woefully incomplete. While we have made major progress in understanding some aspects of pH regulation during phagosome maturation, we are just beginning to appreciate the existence and importance of phagosome resolution and know little about pH regulation at this stage. Also, as pathways connecting phagocytosis, microbial survival within phagosomes, and metabolic reprogramming in both host and pathogen are progressively revealed, the role of pH in these processes needs to be evaluated in detail. Indeed, metabolite transport and utilization are both exquisitely pH sensitive events. As such, the establishment and regulation of the luminal pH should remain a central component of future studies of host-pathogen interactions.

Lastly, it is worth emphasizing that past studies on pH regulation have been essentially limited to *in vitro* and *ex vivo* studies using isolated cells. We anticipate that advances in intravital imaging will extend these analyses to more complex, physiological settings.

## Author Contributions

JW and SG wrote and critically reviewed the manuscript. Both authors contributed to the article and approved the submitted version.

## Conflict of Interest

The authors declare that the research was conducted in the absence of any commercial or financial relationships that could be construed as a potential conflict of interest.
